# MRI features of an atypical case of extraventricular neurocytoma: A case report

**DOI:** 10.1097/MD.0000000000028207

**Published:** 2021-12-23

**Authors:** Xiaoge Liu, Yaohan Yu, Liwei Ma, Jingliang Cheng

**Affiliations:** a Department of Magnetic Resonance Imaging, the First Affiliated Hospital of Zhengzhou University, Zhengzhou, China; b Department of Radiology, the Children's Hospital of Zhejiang University School of Medicine, Hangzhou, China; c Department of Clinical Laboratory, the First Affiliated Hospital of Zhengzhou University, Key Clinical Laboratory of Henan Province, Zhengzhou, China.

**Keywords:** extraventricular neurocytoma, magnetic resonance imaging

## Abstract

**Rationale:**

Central neurocytoma occurring outside the ventricles is known as extraventricular neurocytoma (EVN). EVN is rare and its magnetic resonance imaging (MRI) findings vary greatly and overlap with the imaging features of other intracerebral primary tumors.

**Patient concerns:**

A 21-year-old woman with an intrauterine pregnancy of 18+2 weeks complained of dizziness and headache for 3 months.

**Diagnosis:**

A 8.6 cm × 5.8 cm × 3.7 cm space-occupying lesion was seen in the right frontal lobe on MRI, with mixed long signals on T1-weighted imaging and mixed slightly long signals on T2-weighted imaging, slightly hyperintense on T2-weighted imaging fluid attenuated inversion recovery images, and a large-scope long T1-weighted imaging and long T2-weighted imaging cystic component at the center of the lesion. A thick fence-like enhancement of the solid component at the periphery of the lesion was observed after injecting a contrast medium, while the internal cystic component was not enhanced. The MRI diagnosis was of glioma. The lesion was pathologically confirmed as an atypical central neurocytoma of the right frontal lobe.

**Interventions:**

Resection of the lesion and postoperative radiotherapy.

**Outcomes:**

The patient was lost to follow-up.

**Lessons:**

EVN can be considered as a differential diagnosis for lesions occurring in the cerebral hemispheres of young patients with cystic degeneration, thick fence-like enhancement, and peritumoral edema on MRI.

## 1 Introduction

Central neurocytoma (CN) is a rare type of neuroepithelial tumor that occurs in the region of the foramen of Monro in the lateral ventricles in young adults.^[1]^ CN occurring outside the ventricles is known as extraventricular neurocytoma (EVN), and the annual incidence of EVN is 0.009/100,000.^[2]^ Compared with CN, EVN has a relatively poor prognosis.^[3,4]^ EVN and other intracranial tumors have overlapping imaging and histological features, making it difficult to diagnose.^[5]^ We report a case of atypical EVN in a 21-year-old pregnant woman, which was confirmed by surgical pathology.

## 2 Case presentation

A 21-year-old woman with an intrauterine pregnancy of 18+2 weeks experienced dizziness and headache 3 months ago, with aggravation at night, accompanied by blurred vision and decreased visual acuity. Nausea, vomiting, and poor limb mobility, were not observed. Subsequently, the symptoms aggravated, and cranial magnetic resonance imaging (MRI) at a local hospital showed a space-occupying lesion in the right frontal lobe, considered as glioma. The patient was then admitted to the oncology department of our hospital.

**Figure 1:**
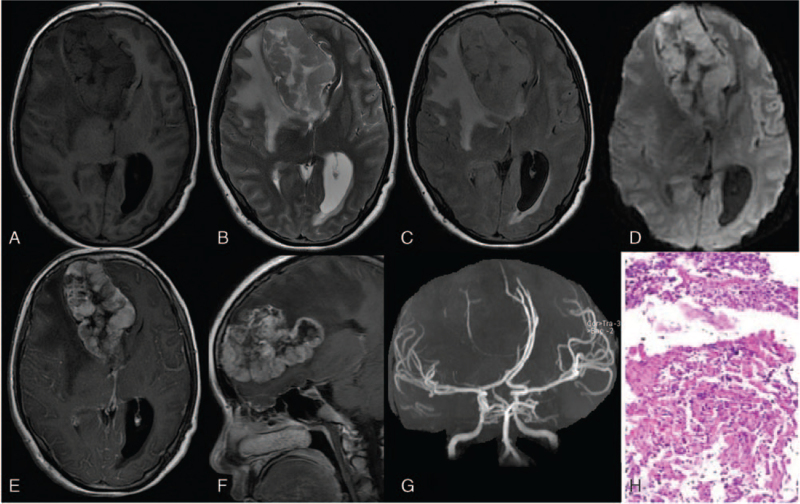
Magnetic resonance imaging and pathological images of atypical extraventricular neurocytoma in the right frontal lobe of a 21-year-old woman. In Figure 1A, axial T1-weighted image shows an irregular mixed signal lesion in the right frontal lobe. Compared with the gray matter, the peripheral part shows an isointense signal, and the central part shows a hypointense signal. In Figure 1B, axial T2-weighted image reveals that, compared with the gray matter, the peripheral part of the lesion shows an isointense signal, while the central part shows a significant hyperintense signal. A large edematous region with a long T2 signal is seen around the lesion, and the midline structures appear to have shifted to the left. In Figure 1C, according to the axial T2-weighted image fluid attenuated inversion recovery (FLAIR), the lesion has a slightly mixed hyperintense signal. In Figure 1D, the high b value of diffusion weighted image shows that the peripheral part of the lesion has a slightly hyperintense signal. In Figure 1E, axial contrast enhanced T1-weighted image reveals a significant thick fence-like enhancement in the peripheral part of the lesion while there is no enhancement in the central part. In Figure 1F, sagittal enhanced T1-weighted image shows that the lesion is lobulated. In Figure 1G, brain magnetic resonance angiography shows a leftward shift of the bilateral anterior cerebral arteries and a posterior shift of the right middle cerebral artery, both under compression. Figure 1H, H&E (×400) staining shows that the mass is composed of uniform small round cells.

Plain MRI revealed a large space-occupying lesion in the right frontal lobe, with mixed long T1 and mixed slightly long T2 signals, slightly hyperintense T2 fluid attenuated inversion recovery signal, as well as a large long T1 and long T2 cystic component at the center of the lesion (Fig. 1A–C). The solid component showed slight hyperintensity on diffusion-weighted imaging (Fig. 1D) and slight hypointensity on the apparent diffusion coefficient images. It was approximately 8.6 cm × 5.8 cm × 3.7 cm in size. There was significant edema around the lesion, the falx cerebri and corpus callosum were compressed, and the midline structure showed a leftward shift. The anterior horn of the right lateral ventricle was compressed, and its structure was unclear. Contrast-enhanced MRI showed significant thick fence-like enhancement of the solid component at the periphery of the lesion, while the internal cystic component was not enhanced (Fig. 1E, F). The boundary of the lesion was clear and peripheral edema was not enhanced. Brain magnetic resonance angiography showed that the bilateral anterior cerebral arteries had shifted to the left, and the right middle cerebral artery had shifted posteriorly under compression (Fig. 1G).

After admission and completion of the examinations, the patient was administered treatment for decreasing the elevated intracranial pressure. Considering her disease condition, the patient and her family strongly demanded transferring the patient to the obstetrics department for induced labor. Ultrasound-guided amniocentesis was performed to induce labor. After the operation, the patient was transferred to the neurosurgery department and underwent resection of the brain space-occupying lesion under general anesthesia. During the operation, the tumor showed an irregular shape, no complete capsule, an unclear boundary with the surrounding brain tissue, and occupied most of the right frontal lobe, with a significant occupying effect. The falx cerebri was squeezed to the left, and the tumor tissue was resected piecemeal and clamped under a microscope. The tumor was pathologically confirmed as an atypical CN of the right frontal lobe (equivalent to WHO Class II-III) (Fig. 1H). The following immunohistochemistry findings were observed: Oligo-2(−), CD34 (vascular+), CD56 (+), Syn (+), GFAP (partial+), CK (−), EMA (−), Ki-67 (30%), and Neu-N (−). The patient underwent postoperative radiotherapy (56 Gy). The patient did not return to the hospital for review after radiotherapy. The follow-up was not achieved.

## 3 Discussion

In 1997, Giangaspero et al first described EVN, the biological behavior and histological characteristics of which are similar to those of CN.^[2]^ In 2007, EVN was classified as an intracranial WHO Class II tumor, and in 2016, it was classified as a neuronal and mixed neuronal-glial tumor according to the WHO classification.^[2]^ Compared with CN, EVN has a relatively poor prognosis. EVN frequently occurs in children and young people, and the age of onset is 5 to 76 years (median age: 34 years). There is no difference in the incidence between men and women. EVN frequently occurs in the frontal lobe, followed by the temporal and parietal lobes, and some cases in the sellar region, spinal cord, and other sites have been reported.^[3]^ The clinical manifestations of EVN are related to the location of the tumor and compression of the surrounding tissues and include epilepsy, headache, and impaired vision.^[4]^ EVN histologically presents as uniform small round cells with neuronal differentiation, transparent cytoplasm embedded in the neuropil, and a strong immune response to synaptophysin.^[3]^ EVN with an MIB-1 labeling index >3% and atypical histology is known as atypical EVN. Atypical histological features include necrosis, microvascular hyperplasia, infiltration of surrounding tissues, and increased mitosis. An increase in the MIB-1 labeling index indicates a higher proliferation potential, while atypical histological features indicate malignant features, both of which suggest a relatively poor clinical outcome.^[3]^ Gross total resection is the mainstay of therapy, adjuvant modality includes radiation therapy, chemotherapy. EVN is associated with bad prognosis. Rades et al^[6]^ found overall 5-year recurrence rate was 68%.

Due to the different locations of cell variation and pathological changes, the imaging findings of EVN differ.^[4]^ Usually, the imaging manifestations are of cystic and solid lesions with clear boundaries in the cerebral hemispheres, involving the gray matter and deep white matter. A study by Xiong et al^[3]^ showed that half of the patients had peritumoral edema. Romano et al^[2]^ showed that calcification can occur in more than 50% of lesions. Ganglion differentiation makes EVN prone to cystic degeneration.^[5]^ On MRI, the solid component of EVN shows isointense signal on T1-weighted images (compared with gray matter) and mixed signals on T2-weighted images, which are mainly isointense and slightly hyperintense signals. The cystic component shows hypointensity on T1-weighted images, T2-weighted fluid attenuated inversion recovery images, and hyperintensity on T2-weighted images. The calcification component shows hypointensity on T1-weighted images and T2-weighted images. After enhancement, it shows mild to severe heterogeneous enhancement. In some reported cases, EVN showed annular, focal, and zebra-like enhancement,^[4]^ and some researchers observed significant block-like and nearly homogeneous enhancement of the lesions.^[7]^ No enhancement was observed in some cases.^[4]^ Romano et al^[2]^ showed that atypical EVN had more invasive imaging features, characterized by a relatively large volume (usually larger than 5 cm in diameter), cross-lobe growth, lobulation, unclear boundaries, flow void in blood vessels, necrosis, cystic degeneration, and hemorrhage. In the present case, there was a huge cystic and solid lesion in the right frontal lobe with lobulated changes (lobulation was clearly displayed in the sagittal sections [Fig. 1F]), with significant space-occupying effect and peritumoral edema. On enhancement, it showed significant peripheral enhancement, mainly characterized by a thick fence-like enhancement and relatively sharp edges; the remaining portion showed line-like enhancement. The cystic component in the lesion was not enhanced, and no enhancement was found in the surrounding edematous area. The imaging features are consistent with those reported in the literature, with a large volume of the lesion and lobulated changes, consistent with the features of atypical EVN. However, the thick fence-like enhancement after contrast infusion has not been mentioned in the literature, which also proves that the imaging features of EVN vary greatly.

EVN should be differentiated from the following diseases: oligodendroglioma, high-grade astrocytoma, and extraventricular ependymoma. Oligodendroglioma frequently occurs in middle-aged patients. The lesions originate from white matter, can break outward through gray matter, and can also involve the skull bone (this sign supports the diagnosis of oligodendroglioma rather than EVN).^[7]^ The typical MRI features of the lesions include hypointensity on T1-weighted images and hyperintensity on T2-weighted images as compared to gray matter, and 70% to 90% of patients can have macrocalcification (calcification in EVN is mostly punctate). T1 after contrast infusion shows that 15% to 20% of cases demonstrate enhancement with an unclear boundary. High-grade astrocytoma frequently occurs in patients aged 40 to 50 years. MRI shows isointensity or hypointensity on T1-weighted images and isointensity or hyperintensity on T2-weighted images. It is usually more invasive, accompanied by a space-occupying effect and edema around the tumor, and calcification is rare.^[7–9]^ Extraventricular ependymoma is rare and usually large (>4 cm). MRI can show significant cystic degeneration, and the lesions show small patchy solid enhancement after contrast infusion. Calcification is common and is mostly linear, punctate, or irregular. In some cases, “Catharanthus roseus”-like signs, which refer to centripetal calcification at the edges and necrosis in the central area, may appear on plain computed tomography.^[7]^

EVN is rare, and the MRI signals vary greatly and overlap with the imaging features of other intracerebral primary tumors. Therefore, when lesions occur in the white matter area of the cerebral hemispheres of young patients, accompanied by calcification, cystic degeneration, significant enhancement, peritumoral edema, and other signs, EVN can be considered in the differential diagnosis. The final diagnosis depends on the pathology findings.

## Author contributions

**Conceptualization:** Xiaoge Liu.

**Investigation:** Xiaoge Liu.

**Supervision:** Jingliang Cheng.

**Writing – original draft:** Xiaoge Liu.

**Writing – review & editing:** Yaohan Yu, Liwei Ma.
